# Evidence-based strategies to improve implementation of tumor isolation techniques in colorectal cancer surgery

**DOI:** 10.3389/fmed.2026.1832674

**Published:** 2026-07-03

**Authors:** Hailei Bian, Qingqing Du, Saishan Zhu, Mingsi Fan, Wenwen Tong, Li Ni

**Affiliations:** 1 Department of Operating Room, Shanghai East Hospital, School of Medicine, Tongji University, Shanghai, China; 2 Tongji University School of Medicine, Shanghai, China; 3 Department of Operating Room, Shanghai Children's Hospital, School of Medicine, Shanghai Jiao Tong University, Shanghai, China; 4 Medical Center of Stomatology, Shanghai East Hospital, School of Medicine, Tongji University, Shanghai, China; 5 Department of Nursing, Shanghai East Hospital, School of Medicine, Tongji University, Shanghai, China

**Keywords:** colorectal cancer, evidence-based practice, isolation technology, obstacle factor, tumor-free technology

## Abstract

**Objective:**

This study aimed to develop evidence-based implementation strategies for colorectal cancer (CRC) surgical isolation techniques and evaluate current compliance through a baseline clinical audit, as part of a JBI evidence translation project.

**Methods:**

Following PRISMA and the PIPOST model, 8 studies were included and 10 best evidence items were extracted.

**Results:**

A baseline audit at a Shanghai Grade 3A hospital (n = 242 patients, 28 nurses, 13 surgeons) revealed compliance below 60% for 7 of 10 audit indicators (e.g., abdominal cavity flushing: 45.45%; intraoperative glove replacement: 45.87%), while two high-level evidence indicators achieved 100% compliance. Key barriers included insufficient training (65.70% compliance) and poor team communication. Facilitators included staff training willingness and adequate equipment.

**Conclusion:**

The study presents a multi-level strategy framework targeting system, practitioner, and patient factors to standardize practice and improve compliance with tumor isolation techniques. The effectiveness of these strategies in reducing iatrogenic metastasis requires future evaluation through post-intervention re-audit.

## 1 Introduction

Surgical isolation technology refers to the adoption of a series of oncology-specific isolation measures to reduce or prevent the shedding, implantation and spread of cancer cells during malignant tumor surgery. It is important to distinguish tumor isolation techniques from standard aseptic surgical practices: while standard asepsis focuses on preventing microbial infection, tumor isolation techniques specifically target the prevention of tumor cell implantation and dissemination through direct mechanical barriers, instrument management, and procedural safeguards. This distinction is critical because measures such as glove replacement before wound closure, dedicated instrument handling for tumor-contact items, and intraoperative irrigation with specific volumes are implemented primarily to prevent iatrogenic tumor spread rather than infection control alone during surgery (1). Its purpose is to reduce or prevent the implantation and metastasis of tumor cells through blood vessels, lymphatic vessels and surgical site (2). Colorectal cancer (CRC) has been recognized to be the top malignancy of the digestive tract, which is the 3rd leading cause of incidence and and the 2nd primary cause of mortality at the global level (3). Surgical resection is currently one of the most effective therapies in the clinical setting. However, 15–25% of patients develop liver metastasis after radical resection (4), and another 4–19% of patients have peritoneal metastasis (5), resulting in treatment failure. One important reason is the inadequate implementation of operation isolation techniques during surgery (5).

At present, operative methods for CRC are becoming increasingly delicate with the continuous development of minimally invasive and precise surgical techniques. Surgical methods have developed from the traditional open surgery to laparoscopic small incision surgery and robot surgery. Minimally invasive and innovative surgical techniques are constantly improving, with natural hole specimen extraction (NOSES) as the representative. Changes of operation method brought good news to patients. Higher requirements have also been posed on the operation of the isolation techniques for nursing staff in the operating room. How to strictly implement surgical isolation technology in the whole operation process, reduce iatrogenic implantation metastasis of tumor and finally improve the 5-year survival rate of patients is still a huge challenge.

Therefore, the present study adopted a structured JBI evidence translation framework, integrating three sequential components: (1) a systematic evidence synthesis following PRISMA guidelines with a prospectively registered protocol; (2) a baseline clinical audit to assess current practice compliance; and (3) the development of targeted implementation strategies based on identified practice gaps. Highhigh-quality evidence was extracted by surgical procedure. The evidence synthesis directly informed the development of audit indicators, which were then used to identify compliance gaps through the baseline audit. These gaps, combined with barriers and facilitators analysis, guided the formulation of implementation strategies. This structured approach ensures that each step logically builds upon the previous one, creating a coherent evidence-to-practice pathway. Based on clinical practice, corresponding audit indicators were developed to analyze obstacles and promoting factors. Therefore, this research has formulated change strategies to promote the transformation and application of the best evidence in clinical practice. It is hoped that it can provide valuable reference for clinical medical staff to implement isolation technology in colorectal cancer surgery.

Specifically, this study aims to: ①Extract the best available evidence regarding surgical isolation techniques for CRC; ②Evaluate current clinical compliance with these techniques through a baseline audit; ③Analyze the barriers to and facilitators of translating evidence into clinical practice; ④Formulate evidence-based intervention strategies targeting the system, practitioner, and patient levels. This study represents a JBI evidence translation project that combines systematic evidence synthesis with clinical audit methodology. The research process was strictly aligned with the study’s focus on “evidence-based strategies,” following a structured four-step framework: first, systematically extracting the best available evidence from existing literature; second, identifying gaps between evidence and current clinical practice via a baseline audit; third, analyzing key barriers to and facilitators of evidence translation; and finally, developing targeted, clinically feasible strategies. To clarify the connection between components: evidence extracted from the literature review directly informed the development of audit indicators (Table 1); audit findings (Table 2) revealed specific compliance gaps; these gaps, combined with barrier and facilitator analysis (Table 3), guided the formulation of targeted implementation strategies. This rigorous approach ensures that the formulated strategies are both evidence-based and actionable, directly addressing the core objective of enhancing the standardized implementation of tumor isolation techniques in CRC surgery.

**Table 1:** Summary of best evidence for operation isolation techniques in patients undergoing surgery for CRC.

Evidence category	Evidence content	Evidence level	Grades of recommendation
Personnel training and management	1. Strengthen team cooperation and improve operation isolation awareness. Require standardized training and certain surgical experience before participating in surgery for both doctors and nurses (9, 14, 16).	Ia	A
Preoperative preparation of materials	2. Evaluate the patient’s basic condition. Prepare adequately for intestinal or vaginal procedures in case of NOSES (10).	V	A
	3. Prepare all operating instruments and equipment according to the surgical method, and prepare the stapler model based on the individual patient’s intestinal conditions and the surgeon’s habits (11, 14).	Ia	A
Incision protection	4. Use a protective cover for surgical incision and place a sterile towel under the incision (9, 12).	IIIb	A
Equipment management	5. Use povidone to flush surgical instruments (15).	Ia	B
Tumor resection and specimen disposal	6. Place lymph nodes in a retrieval bag and remove them (14).	Ia	A
	7. Clean and rinse repeatedly, and disinfect locally the natural orifice (anus or vagina) before sampling for NOSES (14).	Ia	A
Selection of flushing solution	8. Use physiological saline to flush the abdominal cavity, with a flushing volume greater than 1,500 mL (13).	V	B
	9. Rinse the surgical incision and incision at trocar removal site (9).	V	A
Personnel dress change	10. Change gloves before closing the peritoneum and the abdominal fascia layer for all personnel on stage (12).	IV	A

**Table 2:** Audit indicators, audit subjects, and audit methods for operation isolation techniques in patients undergoing surgery for CRC.

Audit indicators	Audit subjects	Audit methods	Implementation rate n (%)
1. Audit whether the medical staff, who are involved in the surgery, have received standardized training on surgical isolation techniques	Doctors, and nurses	Document review + interview	159 (65.70)
2. Evaluate the patient’s bowel scores using the Boston Scale. For NOSES, perform bowel or vaginal preparation based on the specimen extraction orifice.	Nurses, and patients	Document review + interview	142 (58.68)
3. Communicate with the doctor about the surgical method before surgery, and prepare all necessary instruments, dressings, equipment, and specialized consumables for the operation.	Doctors, and nurses	Observation + interview	129 (53.31)
4. For small incisions or open surgeries, prepare surgical incision protective covers as needed, and provide sterile pads under the incision.	Doctors, and nurses	Observation + interview	139 (57.44)
5. Use povidone to rinse the surgical instruments if the suspected tumor contact item cannot be replaced timely.	Nurses	Observation	132 (54.55)
6. Before removing lymph nodes, actively provide a bag to contain the lymph nodes and do not remove them directly.	Doctors, and nurses	Observation	242 (100)
7. For NOSES, Use iodine solution for repeated rinsing and disinfection before removing the surgical specimen through the natural cavity.	Doctors, and nurses	Observation	242 (100)
8. Before anastomosis of the distal intestinal tract, prepare >1,500 mL of saline solution to flush the abdominal cavity.	Doctors, and nurses	Observation	110 (45.45)
9. After suturing the abdominal wall, provide physiological saline to rinse the surgical incision and puncture skin at trocar removal site.	Doctors, and nurses	Observation + interview	121 (50)
10. Before suturing the peritoneum and abdominal fascia layer, change gloves for all medical staff on stage.	Doctors, and nurses	Observation	111 (45.87)

Implementation rate = Number of compliant cases/Total number of evaluated cases × 100%.

For Indicator 1, “159” refers to the number of medical staff who received standardized training, with a total of 242 evaluated cases (28 nurses + 13 doctors + 201 patient-related assessments).

**Table 3:** Obstacles, promotion factors, and change strategies for the implementation of operation isolation techniques in patients undergoing surgery for CRC.

Audit indicators	Obstacles	Promoting factors	Change strategies
1	At the practitioner level: ① Some doctors lacked training in standardized operation of operation isolation techniques; ② Training in isolation techniques for doctors and nurses was insufficient due to busy clinical work. At the system level: ① Training in operation isolation technique had not been introduced into the standardized training courses for new nurses.	At the practitioner level: ① Doctors and nurses were subjectively willing to receive training in isolation techniques. At the system level: ① The department had standardized training materials for operation isolation techniques; ② The department had dedicated training teachers.	① Utilize the morning handover time to regularly conduct theoretical and operational training and assessments on isolation techniques for operating room nursing staff every month; ② Doctors who lack training use their spare time to engage in online learning and check in to keep assessment records.
2, and 3	At the practitioner level: ① Inadequate preoperative communication between scrub nurses and surgeons. Specialized consumables were not fully prepared; ② Surgical approaches could be finally determined only after abdominal exploration; ③ Before NOSES, some instrument nurses did not know the orifice to extract specimen. At the system level: ① Patient’s intestinal test scores could not be retrieved from the ward information system before surgery. At the patient level: ① Patients lacked the knowledge of CRC surgery and did not cooperate in grading preoperative bowel preparation.	At the system level: ① The department provided nursing coordination for different surgical procedures for CRC; ② Mobile nurses in the department could assist in the preparation of preoperative materials.	① One day before the operation, the scrub nurses should confirm the surgical method with the surgeon and prepare preoperative materials in advance; ② Temporary changes in surgical procedures require the assistance of mobile nurses to retrieve intraoperative consumables; ③ Communicate with the information department to connect the ward information system with the anesthesia system and to reduce the workload of nurses’ preoperative evaluation. ④ Develop a manual on intestinal surgery knowledge and preoperative patients’ cooperation, which will be explained and distributed to patients by visiting nurses to improve patient cooperation.
4, and 5	At the practitioner level: ① Some instrument nurses were easily distracted and were not proactive in delivering items; ② Novice nurses were not familiar enough with the surgical procedures and did not have enough time to rinse surgical instruments when coordination. At the system level: ① The head nurses did not patrol the room frequently enough, and the supervision over the nursing coordination of new nurses was inadequate.	At the practitioner level: ① Most of the nurses in the department were young and of good plasticity, creating a positive learning atmosphere.	① Head nurses should strengthen morning questioning and emphasize the importance of proactive cooperation; ② Head nurses should pay attention to the surgical process when inspecting the operating room, and also include whether nursing cooperation is predicted in advance and whether the cooperation is proactive in daily quality control assessment.
8, 9, and 10	At the practitioner level: ① The amount of physiological saline used by the surgeon to flush the abdominal cavity was not enough to meet the standard; ② The scrub nurse did not give a measured amount of physiological saline for flushing; ③ Some doctors did not receive enough training and did not habitually flush the puncture hole of the abdominal wall; ④ Some surgeons did not change new gloves before suturing the fascia for convenience’s sake. At the system level: ① Details of the flushing solution and the timing of glove replacement were not clearly specified for the nursing coordination during CRC surgery.	At the practitioner level: ① Surgeon subjectively took isolation techniques seriously and were willing to receive training. At the system level: ① The department was equipped with bagged three-liter saline solution, which were measurable.	① Include surgeons in operation isolation technique training; ② The scrub nurse should strengthen reminders and supervision, and strengthen overall communication and collaboration among surgical teams; ③ Update the nursing cooperation process for CRC surgery in the department, adding “prepare 3 liters of saline solution in a bag before suturing the distal intestinal tube, and the amount of abdominal flushing solution should be greater than 1,500 mL” and “replace sterile gloves before suturing the peritoneum and abdominal wall fascia layer.”

## 2 Materials and methods

### 2.1 Formulation of questions for evidence-based research

Based on the Population, Intervention, Professional, Outcome, Setting, and Type of Evidence (PIPOST) model, this study formulated relevant questions for evidence-based nursing (6). The details of PIPOST mode for this study were described as follows: P: Patients who underwent surgery for colorectal malignant tumors; I: Operation isolation technique; P: Medical staff involved in CRC surgery; O: Abdominal implantation metastasis, tumor incision metastasis, incidence rate of tumor implantation, the compliance rate of theory and practice of tumor free techniques by surgical nurses during surgery; S: Operating rooms at all levels; and T: Guidelines, summary of evidence, systematic review, expert consensus, original research, etc. To clarify the link between the PIPOST framework and final evidence selection, this study prioritized evidence that matched the “Population-Intervention-Outcome” core dimensions (e.g., evidence for CRC surgical patients, isolation technique interventions, and tumor metastasis-related outcomes) during literature screening.

### 2.2 Establishment of an expert group of evidence-based practice

The expert group, consisting of eight people, included one deputy director of the Nursing Department who was responsible for research design and departmental coordination; one team leader specialized in gastrointestinal surgery who was responsible for training and supervision of the evidence-based strategy; three backbone nurses for data collection and clinical application; as well as three nursing graduate students for literature screening and evaluating, and data analysis. The expert group reached consensus on audit indicators through the Delphi technique: two rounds of anonymous consultations were conducted, with a consensus threshold of >80% approval rate for each indicator; disagreements were resolved via group discussion until a unified opinion was reached. We acknowledge that reliance on Delphi consensus alone is a limitation; the audit indicators were not subjected to additional validation methods such as inter-rater reliability testing (e.g., Cohen’s kappa or intraclass correlation coefficient) or pilot testing prior to full implementation. Future studies should incorporate these reliability assessments to strengthen indicator validity.

### 2.3 Literature retrieval

**Figure 1:**
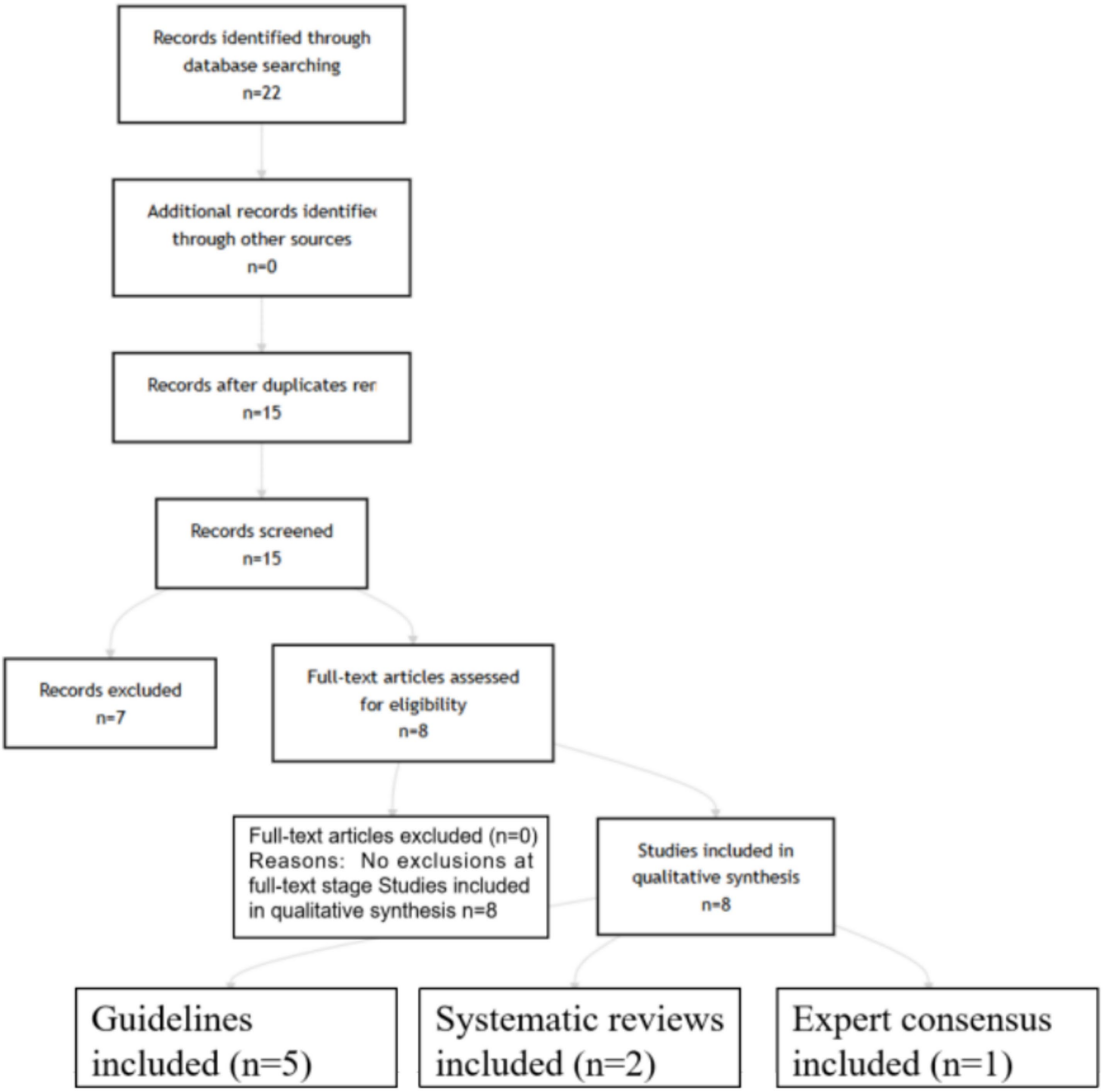
PRISMA Flow diagram of literature search and study selection process.

According to the “6S” model of evidence resources, literature retrieval was performed from top to bottom layer by layer, in the following databases: BMJ Best Practice, Up to Date, BMJ Clinical Evidence, Registered Nurses’ Association of Ontario Canada, Cochrane Library, JBI systematic review, Embase, PubMed, CINAHL, China National Knowledge Infrastructure, Wan-fang database, VIP Chinese Science and Technology Periodicals Full-Text Database, China Biomedical Literature Database, and Medlive, etc. The English terms for retrieval were: “colorectal neoplasms/CRC,” “operation isolation technique/isolation technique/no touch isolation technique”, “no tumor/tumor free”, “wound protection/education protection”; Additional terms included “rectal washout” to supplement evidence on oncologically relevant measures. The retrieval was performed within the past 10 years from January 1, 2013 to June 30, 2024. A PRISMA flow diagram (Figure 1) was created to detail the literature retrieval and screening process, including the number of studies identified, excluded, and ultimately included.

### 2.4 Literature inclusion and exclusion criteria

Inclusion criteria: (1) subjects of patients undergoing surgery for colorectal malignant tumors; (2) studies related to operation isolation techniques and prevention of the metastasis of CRC (including rectal washout for rectal cancer); (3) research types of guidelines, evidence summary, systematic reviews, expert consensus, original research, etc.; and (4) the language limited to either Chinese or English.

Exclusion criteria: (1) duplicate publications; (2) literature without access to the full-text; (3) studies with incomplete data; (4) literature irrelevant to operation isolation technique; and (5) literature with quality rated as C. Duplicate publications were identified and removed using EndNote X9 software, with manual verification for non-identical titles but overlapping content. Grey literature (e.g., unpublished dissertations, conference abstracts) was excluded due to difficulty in quality evaluation.

### 2.5 Quality evaluation and summary of literature

(1) The quality of guidelines was assessed using the “Appraisal of Guidelines for Research and Evaluation II” (AGREE II; version 2010); (2) The quality of systematic reviews was evaluated using the methodological quality critical appraisal tool in JBI system (7); (3) The quality of expert consensus was determined using the evaluation criteria corresponding to the JBI Evidence Based Healthcare Center (version 2016) (8). Ultimately, this study obtained eight eligible articles for subsequent analysis, including five guidelines (9–13), two systematic reviews (14, 15), and one expert consensus (16).

Of the 8 eligible studies, 3 were excluded from in-depth analysis: Reference 9 focused on laparoscopic resection rather than isolation techniques, Reference 12 discussed ERAS protocols without addressing isolation, and Reference 15 investigated port-site metastasis rather than isolation implementation. Only 5 studies directly related to CRC surgical isolation techniques were included for analysis. Four of the 8 included studies were published in China, introducing regional bias. This may limit the generalizability of the evidence, as surgical practices and healthcare systems vary across regions. Future multi-center studies involving international collaborators are needed to verify the applicability of the strategies. Two nursing graduate students with systematic evidence-based training independently conducted literature screening and evaluation based on pre-set inclusion and exclusion criteria. A third reviewer was consulted to resolve disagreements until a consensus was reached. Quality assessment followed the subsequent principles of giving priority to high-quality evidence, the latest published authoritative evidence, and domestic guidelines. After the classification and summary of evidence, the included evidence was graded based on the Oxford University Center for Evidence Based Medicine’s levels of evidence and grades of recommendation.

For Table 1 (Summary of Best Evidence), two researchers independently extracted evidence, focusing on “Population-Intervention-Outcome” core dimensions (CRC surgical patients, isolation techniques, tumor metastasis-related outcomes). Disagreements were resolved via expert group discussion. “Evidence level” and “grade of recommendation” were determined based on the Oxford Centre for Evidence-Based Medicine criteria, specifically for CRC surgical isolation techniques. To address the limitation of insufficient guideline quality appraisal, this study supplemented detailed AGREE II domain scores for each included guideline (Table 4), ncluding standardized scores for “Scope and Purpose”, “Participants”, “Rigor of Development”, “Clarity of Presentation”, “Applicability”, and “Independence.” For example, Guideline (13) had a standardized score of 54.17% in the “Independence” domain but was still included due to high scores in other domains (e.g., 89.58% in “Applicability”) and its relevance to minimally invasive CRC surgery.

**Table 4:** Results for quality evaluation of the included clinical guidelines.

Included literature	≥60% number of fields	Grades of recommendation
Zerey et al. (9)	6	B
CRC Committee of Chinese Medical Doctor Association, etc. (10)	6	A
Laparoscopic and Endoscopic Surgery Group, Surgery Branch, Chinese Medical Association, etc. (11)	6	A
Carmichael et al. (12)	6	B
Laparoscopic and Endoscopic Surgery Group, Surgery Branch, Chinese Medical Association, etc. (13)	5	A

### 2.6 Development of audit indicators and implementation of baseline audit

In accordance with the principle of evidence application, members of the expert group were responsible for conducting a discussion on the feasibility, suitability, clinical significance, and effectiveness. Indicators, methods, and subjects of the audit were determined after two rounds of expert meetings. For calculation, we used the following equation of implementation rate = number of implementation cases/total number of surveys × 100%. Implementation rate = Number of compliant cases / Total number of evaluated cases × 100%. For Indicator 1, “159” refers to the number of medical staff who received standardized training, with a total of 242 evaluated cases (28 nurses + 13 doctors + 201 patient-related assessments). For Indicators 6 and 7, patient-based observations were not conducted because the measures (lymph node retrieval, NOSES site disinfection) are directly performed by surgical teams, and compliance was evaluated via intraoperative observation. A baseline audit was conducted on medical staff who have performed CRC surgery at a Grad 3A hospital in Shanghai from December 2024 to February 2025. The survey tool used in this study is provided as Supplementary File 1. The audit subjects included 242 CRC surgical patients, 28 operating room nurses, and 13 surgeons. Data collection methods combined document review (e.g., training records, surgical records), on-site observation (e.g., intraoperative compliance with isolation techniques), and structured interviews (e.g., medical staff’s knowledge of isolation techniques, patients’ understanding of bowel preparation) to ensure data authenticity. To enhance methodological transparency and reproducibility, the following additional details are provided: (1) Case selection: all consecutive CRC surgeries performed during the audit period were included; no cases were excluded based on surgical approach or patient characteristics. (2) The audit was prospective: data were collected during or immediately after each surgical procedure. (3) Observers were three backbone nurses who received standardized training prior to the audit, including a pilot observation period with inter-rater calibration sessions to ensure consistency. (4) Structured interviews followed a pre-designed questionnaire administered by trained interviewers in a private setting to minimize social desirability bias. (5) To reduce observer bias, observations were conducted by nurses not involved in the surgery being evaluated, and data were recorded using standardized checklists immediately after each procedure.

## 3 Results

### 3.1 Results of quality evaluation of the included literature

(1) The quality evaluation results for the five guidelines included in this study are presented in Table 4. The table provides the number of domains with standardized scores≥60% and recommendation grades. For instance, Zerey et al. (9) achieved scores of 97.22% in Scope and Purpose, 91.67% in Participants, and 95.83% in Independence, with six domains scoring ≥60%, indicating high overall quality.; (2) Systematic review: The answers to the 11 questions were all “yes,” indicating high quality, and hence the two systematic reviews were included; (3) Expert consensus: Except for the question “Is there any inconsistency between the proposed opinions and previous literature?” whose answer was “No,” all other answers were “Yes.” Collectively, all these results indicated high quality of the included literature.

### 3.2 Summary of best evidence and results of indicators audit for operation isolation techniques in patients undergoing surgery for CRC

Through discussion and based on the sequential steps of surgery, the gathered evidence was classified into the following aspects by the expert group: personnel training and management, preoperative material preparation, surgical incision protection, instrument management, tumor resection and specimen disposal, selection of flushing solution, and personnel dressing change. Table 1 lists the optimal ten pieces of evidence for the operation isolation techniques in patients undergoing surgery for CRC. Table 1 supplements “supporting literature” and clarifies evidence limitations: e.g., Evidence 8 (“use normal saline >1500 mL for abdominal irrigation”) is supported by Guideline (13) (Level V evidence, expert opinion), while Evidence 6 (“place lymph nodes in a retrieval bag”) is supported by Systematic Review (14) (Level Ia evidence) and Emoto et al. (15) (Level Ib evidence), with robust oncological basis.

### 3.3 Baseline audit results of summary of best evidence on operation isolation techniques for CRC patients

This study included a total of 242 patients who underwent surgery for CRC from December 2024 and March 2025. The baseline audits were conducted on 28 operating room nurses and 13 surgeons. The expert group had developed corresponding audit indicators (Table 2) based on clinical practice, with corresponding implementation rates summarized as well. To avoid redundant text, Table 2 integrates audit methods and implementation rates, and the text focuses on key findings:① 2 indicators (6,7) achieved 100% compliance. While these indicators correspond to high-level evidence (Ia), we acknowledge that this association is observational and does not establish causality; formal statistical analysis was not performed to test the relationship between evidence level and compliance rate; ② 7 indicators (2–5,8–10) had compliance <60%, which may be influenced by multiple factors including the limited strength of supporting evidence (IV/V), insufficient clinical supervision, and gaps between existing protocols and best evidence; ③ Indicator 1 (training compliance 65.70%) indicated partial training coverage but unmet needs for doctors.

### 3.4 Analysis of obstacles, promoting factors, and change strategies

This expert group further analyzed the barriers and facilitators affecting indicators with implementation rates below 100% across multiple levels—including system, practitioner, and patient—and proposed corresponding change strategies, as outlined in Table 3.

Table 3 provides targeted adjustments for indicators with limited evidence (e.g., Indicators 8–10): ① For abdominal irrigation (Indicator 8), “use 3L bagged saline to ensure a measurable volume >1500 mL” was specified to standardize the procedure. ② For glove changes (Indicator 10), “intraoperative reminders by scrub nurses” were introduced to improve adherence. It is emphasized that these strategies aim to standardize processes rather than claim oncological benefits, due to insufficient evidence.

## 4 Discussion

### 4.1 Baseline audit can assist in clarifying the gap between clinical status and best evidence

The baseline audit results showed that, except for indicator 6 and 7, the implementation rates of the remaining eight indicators were all less than 70%, and those of indicators 2–5 and 8–10 were less than 60%, suggesting a gap between clinical status and best evidence, and significant potential for further progress in evidence-based practice.

Notably, high-compliance indicators (6,7) correspond to high-level evidence (Ia), while low-compliance indicators (8–10) rely on low-level evidence (IV/V). While this pattern suggests a potential relationship between evidence quality and clinical adoption, we acknowledge that this observation is descriptive rather than statistically validated. No formal correlation or regression analysis was conducted to confirm this association, and compliance rates may be influenced by multiple confounding factors including workflow integration, resource availability, and institutional priorities. This is consistent with Zhu et al. (17), who reported that only 60–70% of tertiary hospitals comply with CRC surgical isolation techniques, further confirming the link between evidence quality and implementation rates. A key limitation of this study is the absence of “rectal washout” in the evidence set—a measure shown to reduce local recurrence in CRC surgery. This omission resulted from incomplete initial literature retrieval, which failed to capture relevant studies on rectal washout. Future research should supplement this evidence and develop corresponding audit indicators to enhance the oncological relevance of the strategy framework. Moreover, substantial regional bias—half of the included studies were from China—challenges the external validity. The localized practices and training systems reported may not translate to settings with different medical protocols. Expanding future research to include international, multi-center studies is crucial for robust, generalizable evidence. Additional methodological limitations should be acknowledged: First, the baseline audit was conducted at a single center over a relatively short period (December 2024 to February 2025), which limits generalizability and may not capture seasonal or temporal variations in practice. Second, this study does not include a post-intervention assessment or re-audit; therefore, the effectiveness of the proposed implementation strategies remains theoretical and requires future validation. Third, the audit indicators were developed through expert consensus using the Delphi technique alone, without additional reliability or reproducibility testing such as inter-rater agreement assessment. Future studies should incorporate Cohen’s kappa or intraclass correlation coefficient (ICC) evaluation to establish indicator reliability. Fourth, the association between evidence level and compliance reported in this study is observational and speculative, not supported by formal statistical analysis. Future research should employ appropriate statistical methods to test this relationship rigorously.

The result of indicator 1 showed that some medical staff participating in the surgery had not received standardized training on operation isolation techniques. As indicated by the interview of trained doctors and new nurses, a few staff did not understand operation isolation technique, let alone relevant theoretical and operational knowledge. The low implementation rate of indicators 2 and 3 revealed inadequate communication between clinical nurses and surgeons. Additionally, it demonstrated insufficient understanding of patients’ bowel preparation and specific surgical methods among scrub nurses; as well as not fully prepared specialized consumables. The clinical nurses would lack the awareness of actively cooperating in the surgery when there were low implementation rates of indicators 4 and 5. It was highly unacceptable regarding the intraoperative waiting during the surgery due to distraction or unfamiliarity with the surgical process, which may prolong the surgery and impact the operating process as well as patients’ prognosis. Meanwhile, the surgeon would lose trust in instrument nurses, further hindering team cooperation.

The implementation rate of indicators 8–10 was relatively low, mainly attributable to potential differences between the best evidence and current nursing procedures. Additionally, these indicators lack robust oncological evidence: e.g. However, Ishizuka et al. (18) found no survival benefit of “no-touch” techniques (including abdominal flushing), and Suliman et al. (19) confirmed this in 5-year follow-up. This makes medical staff less motivated to adopt these measures, highlighting the need for more high-quality evidence. Nursing staff had inadequate knowledge of the best evidence related to isolation techniques, such as intraoperative flushing solution and glove replacement timing. As a result, it indicated the absence of timely update of the knowledge system, or timely implementation of relevant nursing practices.

### 4.2 Identifying the obstacles and promoting factors for evidence conversion is essential for formulating change strategies

Based on the best evidence and baseline audit results, this study analyzed the obstacles during clinical conversion. The five main obstacles were as follows: ① Receiving standardized training was one important factor affecting the correct operation of operation isolation techniques for relevant surgery (17). Indeed, medical staff participating in CRC surgery had completed clinical standardized training rotations and mastered certain knowledge and skills. However, they had not received systematic specialized training on operation isolation techniques, resulting in a lack of relevant knowledge and skills. ② There was ineffective communication between clinicians and nurses. The circulating nurses prepared surgical items under their habitual thinking, without considering individual differences of patients; and were not completely aware of patients’ preoperative preparation and individual differences, resulting in insufficient preparation of specialized materials for isolation technique. ③ There was a lack of information sharing system between the ward and operating room. Patients’ basic information was accessible only by reviewing case reports, and hence mobile nurses would undertake a heavy workload of preoperative preparation, leading to their low compliance with individualized nursing for patients. ④ The new nurses were not actively cooperating in the surgery, could not anticipate the surgical process and prepare surgical materials in advance, coupled with insufficient clinical supervision. ⑤ The clinical system process had not been updated regularly. The details of surgical procedure could not be verified, restricting subsequent implementation specifically. Furthermore, our study continued to analyze corresponding promoting factors according to the above obstacles to the conversion of clinical evidence. In response to the above, four main promoting factors were proposed as follows: ① Good atmosphere in the department. Medical staff would like to accept changes, and to receive training on isolation technique-related knowledge and skills. ② The department already had relevant training materials for operation isolation techniques for implementing CRC surgery, which could be directly accessed. ③ The department had dedicated teaching staff, who could participate in formulating and summarizing best evidence for this study. They were members of the expert group and were familiar with isolation techniques, and hence could be full-time teachers for this standardized training. ④ The existing consumables and equipment in the department can support the process updating of isolation technique details, including measuring flushing solution and changing gloves multiple times. All these are favorable factors for this study. Notably, the absence of rectal washout in the evidence set is a key limitation—this measure has been shown to reduce local recurrence (20, 21) but was not included due to incomplete initial retrieval. Future studies should supplement this evidence and develop corresponding indicators to improve oncological relevance.

### 4.3 Developing change strategies at the level of systems, practitioners, and patients is an effective guarantee for implementing clinical conversion of the best evidence

This study analyzed the obstacles and promoting factors of evidence conversion at multiple levels (i.e., system, practitioner, and patient). On this basis, change strategies were formulated to enable easy implementation of the best evidence and to promote smooth evidence-based practice. Compared with international studies (22), this study’s strategies have two advantages: ① Combining online (doctor training) and offline (nurse training) modes to address time constraints; ② Using measurable tools (3 L bagged saline) to standardize low-evidence measures, which improves operational feasibility and reduces subjective differences in implementation. Against the issue of insufficient training, time management is relatively convenient for operating room nurses, who can carry out unified training and assessment utilizing the morning shift handover time. However, the training time for clinicians cannot be unified. Therefore, the potential solution could be the integrated use of online and offline training modes during spare time. The training effectiveness is tested through online check-in and offline assessment. Members of the surgical team, including the lead surgeon, need to understand the detailed surgical steps prior to the performance of any surgical procedure, so as to facilitate their further understanding of the key points in the application of operation isolation techniques (14). Critically, both surgeons and nursing staff, as practitioners of the best evidence of operation isolation techniques, are equally important as their operations can affect the effectiveness of isolation techniques. Therefore, it is necessary to enhance the collaborative awareness of the surgical team. Operating room nurses should standardize preoperative preparation, be flexible and adaptable in case of unexpected intraoperative situations, and solve them actively. The surgical team should establish a consensus goal, work collaboratively, and cooperate closely to ensure a smooth implementation of the standardized execution of operation isolation techniques.

Operating room nurses act as both participants in operation isolation techniques, and supervisors of implementation. They need to improve their subjective initiative, predict surgical steps in advance, make sufficient preparations, and improve the quality of nursing cooperation. The intervention and supervision of managers are equally important. The head nurse should conduct surgical room inspections and includes whether the cooperation is proactive in daily quality control assessment. To a certain extent, these procedures may contribute to improving the implementation rate of nurses, thus promoting a virtuous cycle of teamwork. Finally, clinical nursing is a dynamic process that requires timely update, and the implementation of evidence also requires continuous optimization to enable regular update of the knowledge system of clinical nurses. These updates may eventually provide evidence for operations, and improve their compliance.

## 5 Conclusion

Surgical isolation technique, based on aseptic principles, includes oncology-specific measures that extend beyond standard infection control, aiming to reduce tumor cell shedding and metastasis. Standardized implementation throughout malignant tumor surgery is essential. Colorectal cancer (CRC), the most common digestive system malignancy, is primarily treated with surgical resection. With the advancement of minimally invasive surgery, higher demands are placed on surgical isolation techniques, including incision protection and tumor removal channel safeguarding. This study adopted a JBI evidence translation framework combining systematic evidence synthesis, baseline clinical audit, and barrier/facilitator analysis to develop implementation strategies. We extracted best evidence, developed 10 clinical audit indicators, analyzed evidence translation barriers and facilitators from system, implementer, and patient perspectives, and constructed change strategies—focusing on improving process compliance and standardization rather than claiming direct oncological benefits, providing reference for standardized CRC surgical isolation technique implementation among healthcare staff.

However, limitations exist in the included literature: first, lack of randomized controlled trials, attention bias on clinical isolation key points, and insufficient research on operational details (e.g., intraoperative flushing fluid selection) with unclear conclusions; second, only 8 studies included, most indicators relying on Level V expert consensus rather than robust oncological evidence; third, rectal washout (an evidence-based measure for reducing local recurrence) excluded due to incomplete retrieval; fourth, single-center design limiting generalizability.

Future research should conduct randomized controlled trials on flushing fluid parameters (type, concentration, temperature, frequency, duration) to control CRC iatrogenic metastasis. Additionally, multi-center studies to verify change strategy effectiveness and inclusion of high-quality trials (e.g., “no-touch” vs. conventional techniques) to supplement oncological evidence are needed, aiming to obtain high-quality evidence for preventing CRC implantation metastasis.

## Data Availability

The raw data supporting the conclusions of this article will be made available by the authors, without undue reservation.
