# Supplementary material for: Evidence-based strategies to improve implementation of tumor isolation techniques in colorectal cancer surgery
Source: Front Med (Lausanne). 2026 Jul 3;13:1832674. doi: 10.3389/fmed.2026.1832674 (PMC13375456; doi:10.3389/fmed.2026.1832674)
Supplement: Supplementary file 1 [file Supplementary_file_1.docx]

**Questionnaire on the Implementation Status of Optimal Evidence for Surgical Isolation Techniques in Patients with Colorectal Malignant Tumors**

**1. Category: Staff Training and Management**

**Evidence Content:** Strengthen teamwork and raise awareness of surgical isolation techniques. Both doctors and nurses must undergo standardized training and possess a certain level of surgical experience before participating in such procedures.

**Audit Indicators:**

1. Possession of a hospital-level or municipal-level operating room competency certificate.

2. Possession of a practicing physician license.

3. Volume of surgical procedures participated in (as a measure of experience).

4. Completion of standardized residency training, including regular participation in hospital course training and passing standardized residency assessments.

**Auditees:** Medical staff (doctors and nurses)

**Audit Method:** Certificate review; System data review; Interviews with medical staff.

**2. Category: Preoperative Preparation of Supplies**

**Evidence Content:** Assess the patient's basic condition. If undergoing a NOSES (Natural Orifice Specimen Extraction Surgery) procedure, ensure adequate bowel or vaginal preparation.

**Audit Indicator:** Nurses use the Boston Bowel Preparation Scale (BBPS) to assess the quality of bowel preparation.

**Auditees:** Nurses / Patients

**Audit Method:** Medical record review / Patient interviews.

**3. Category: Preoperative Preparation of Supplies**

**Evidence Content:** Prepare the necessary operating instruments and equipment according to the surgical approach. Select stapler models based on the patient's intestinal condition and the surgeon's preference.

**Audit Indicator:** Supplies are prepared according to the specialized nursing routines and operational procedures for surgical assistance.

**Auditees:** Medical staff

**Audit Method:** On-site observation; Interviews with medical staff.

**4. Category: Incision Protection**

**Evidence Content:** Use a wound retractor/protector and place a sterile drape under the incision.

**Audit Indicator:** After opening the abdominal cavity, an appropriately sized wound retractor/protector is used based on the incision size, and an additional sterile drape is placed beneath the incision.

**Auditees:** Medical staff

**Audit Method:** On-site observation; Interviews with medical staff.

**5. Category: Instrument Management**

**Evidence Content:** It is recommended to rinse surgical instruments with povidone-iodine solution.

**Audit Indicators:**

1. If instrument quantity is insufficient, it is recommended to clean and disinfect instruments with povidone-iodine solution before reusing them.

2. The pass rate meets the standard rate qualification (achieving the standard) for no-tumor technique handling of instruments is acceptable.

**Auditees:** Nurses

**Audit Method:** On-site observation.

**6. Category: Tumor Resection and Specimen Handling**

**Evidence Content:**

1. Lymph nodes are placed in a retrieval bag for extraction.

2. For NOSES procedures, the natural orifice (rectum or vagina) should be repeatedly cleaned, irrigated, and disinfected locally before specimen extraction.

**Audit Indicators:**

1. Provide appropriate disposable endoscopic specimen retrieval bags, disposable wound protectors, or sterile protective sleeves based on the specimen size and extraction route. Specimen retrieval bags must be changed after each use.

2. For NOSES procedures, the orifice must be irrigated repeatedly with povidone-iodine solution and normal saline until no fecal matter, mucus, or secretions flow out, before inserting the sterile protective sleeve.

**Auditees:** Medical staff

**Audit Method:** On-site observation.

**7. Category: Selection of Irrigation Solution**

**Evidence Content:**

1. Use normal saline for irrigation, with a volume >1500 mL.

2. Irrigate the surgical wound and the trocar removal site incisions.

**Audit Indicators:**

1. After specimen removal and completion of bowel anastomosis, but before drain placement, irrigate the abdominal cavity using >1500 mL of 40°C sterile solution (e.g., normal saline) held in an uncontaminated container.

2. After completing peritoneal closure, provide sterile solution for wound irrigation.

3. According to the extent of tumor metastasis, use chemotherapeutic drugs for intraperitoneal lavage as prescribed.

**Auditees:** Medical staff

**Audit Method:** On-site observation; Interviews with medical staff.

**8. Category: Changing of Attire/Gloves**

**Evidence Content:** All personnel on the surgical field change gloves before closing the peritoneum and before closing the abdominal wall fascia layer.

**Audit Indicator:** Provide appropriately sized gloves for the surgical team to change into before abdominal closure and before closing the fascial layer.

**Auditees:** Medical staff

**Audit Method:** On-site observation.
